# Genomic Surveillance for SARS-CoV-2 Variants: Circulation of Omicron XBB and JN.1 Lineages — United States, May 2023–September 2024

**DOI:** 10.15585/mmwr.mm7342a1

**Published:** 2024-10-24

**Authors:** Kevin C. Ma, Juan Castro, Anastasia S. Lambrou, Erica Billig Rose, Peter W. Cook, Dhwani Batra, Caelin Cubenas, Laura J. Hughes, Duncan R. MacCannell, Paritra Mandal, Neha Mittal, Mili Sheth, Casey Smith, Amber Winn, Aron J. Hall, David E. Wentworth, Benjamin J. Silk, Natalie J. Thornburg, Clinton R. Paden

**Affiliations:** ^1^Coronavirus and Other Respiratory Viruses Division, National Center for Immunization and Respiratory Diseases, CDC; ^2^Office of Advanced Molecular Detection, National Center for Emerging and Zoonotic Diseases, CDC; ^3^Synergy America, Inc., Duluth, Georgia; ^4^Division of Core Laboratory Services and Response, National Center for Emerging and Zoonotic Diseases, CDC.

**Summary**

**What is already known about this topic?**

CDC’s national SARS-CoV-2 genomic surveillance program previously detected the emergence and circulation of major variants, including Delta and Omicron.

**What is added by this report?**

During May 2023–September 2024, SARS-CoV-2 lineages primarily comprised descendants of Omicron XBB and JN.1. Multiple XBB descendants circulated in summer and fall 2023 with immune escape characteristics. JN.1, which was not an XBB descendant, contained substantial genetic differences and became predominant by January 2024. Descendants of JN.1 subsequently emerged. Increases in COVID-19 cases occurred during both variant predominance and cocirculation periods.

**What are the implications for public health practice?**

Given the unpredictable nature of SARS-CoV-2 evolution, continued monitoring for genetic changes and their impact on disease severity and medical countermeasure effectiveness remains essential.

## Abstract

CDC continues to track the evolution of SARS-CoV-2, including the Omicron variant and its descendants, using national genomic surveillance. This report summarizes U.S. trends in variant proportion estimates during May 2023–September 2024, a period when SARS-CoV-2 lineages primarily comprised descendants of Omicron variants XBB and JN.1. During summer and fall 2023, multiple descendants of XBB with immune escape substitutions emerged and reached >10% prevalence, including EG.5-like lineages by June 24, FL.1.5.1-like lineages by August 5, HV.1 lineage by September 30, and HK.3-like lineages by November 11. In winter 2023, the JN.1 variant emerged in the United States and rapidly attained predominance nationwide, representing a substantial genetic shift (>30 spike protein amino acid differences) from XBB lineages. Descendants of JN.1 subsequently circulated and reached >10% prevalence, including KQ.1-like and KP.2-like lineages by April 13, KP.3 and LB.1-like lineages by May 25, and KP.3.1.1 by July 20. Surges in COVID-19 cases occurred in winter 2024 during the shift to JN.1 predominance, as well as in summer 2023 and 2024 during circulation of multiple XBB and JN.1 descendants, respectively. The ongoing evolution of the Omicron variant highlights the importance of continued genomic surveillance to guide medical countermeasure development, including the selection of antigens for updated COVID-19 vaccines.

## Introduction

Approximately 5 years since SARS-CoV-2 emerged, resulting in the global COVID-19 pandemic, Omicron lineages with increased transmissibility and immune escape continue to evolve. CDC has monitored SARS-CoV-2 evolution using national genomic surveillance since December 2020, and variant proportion estimates are updated every 2 weeks on CDC’s COVID Data Tracker.^†^ Data from national surveillance helped guide the selection of XBB.1.5 and JN.1 lineages as the target antigens for 2023–2024 and 2024–2025 COVID-19 vaccines,^§^ respectively, and also supported assessments of potential changes in vaccine and antiviral effectiveness and COVID-19 clinical severity (*1*,*2*). This report summarizes the landscape of Omicron XBB and JN.1 lineage circulation and convergent evolution in the United States during May 14, 2023–September 14, 2024.

## Methods

### Data Sources and Sequence Processing

CDC’s national genomic surveillance program has been previously described (*3*,*4*). CDC integrates SARS-CoV-2 sequence data from 1) the National SARS-CoV-2 Strain Surveillance (NS3) program,^¶^ 2) CDC-contracted commercial laboratories, and 3) public sequence data repositories.** Sequences are then quality-filtered, deduplicated, and assigned Pango lineages.^††^ The median interval from SARS-CoV-2 specimen collection to sequence deposition was 25 days during May 2023–September 2024 (IQR = 22–28 days).

### Estimation of Variant Proportions

Variant proportions for 2-week periods presented on CDC’s COVID Data Tracker were estimated at national and U.S. Department of Health and Human Services (HHS)^§§^ regional levels by specimen collection date; Nowcast estimates for the most recent 4 weeks were not included.^¶¶^ Lineages were included if they constituted ≥1% (unweighted) of sequences nationally and contained spike protein substitutions of potential relevance for vaccines, therapeutics, transmissibility, or severity. Estimates included weighting to account for the complex survey design and potential sampling biases.*** The National Respiratory and Enteric Virus Surveillance System (NREVSS)^†††^ was the primary data source for survey weights beginning November 17, 2023.^§§§^

### Characterization of Lineages

In this analysis, lineages with identical spike residue 31 and receptor binding domain substitutions (residues 332–527) were grouped and denoted as “representative lineage-like”; lineage groups are phylogenetically distinct but have similar spike protein sequences. Normalized frequencies of COVID-19 cases attributable to variants were estimated by multiplying counts of positive test results from NREVSS with variant proportions and scaling by the maximum case count. Sequenced cases were subsampled to assess counts of spike protein amino acid differences (including substitutions, insertions, and deletions).^¶¶¶^ Data were current as of October 11, 2024. This activity was reviewed by CDC, deemed not research, and was conducted consistent with applicable federal law and CDC policy.****

## Results

### Sources of Analyzed SARS-CoV-2 Sequences

A total of 208,357 SARS-CoV-2 sequences from 56 U.S. jurisdictions^††††^ were analyzed from NS3 (1%), commercial laboratories (34%), and public repositories (65%) during May 14, 2023–September 14, 2024. The percentage of sequences from repositories increased from 39% in 2022. The median weekly number of sequences decreased from 21,905 in 2022 to 4,752 in 2023 and 1,989 in 2024 (Supplementary Figure 1, https://stacks.cdc.gov/view/cdc/165772). Previous and updated analytic methods produced similar estimates for XBB.1.5 and HV.1 nationally and regionally (Supplementary Figure 2, https://stacks.cdc.gov/view/cdc/165773).

### XBB Descendants

**FIGURE 1:**
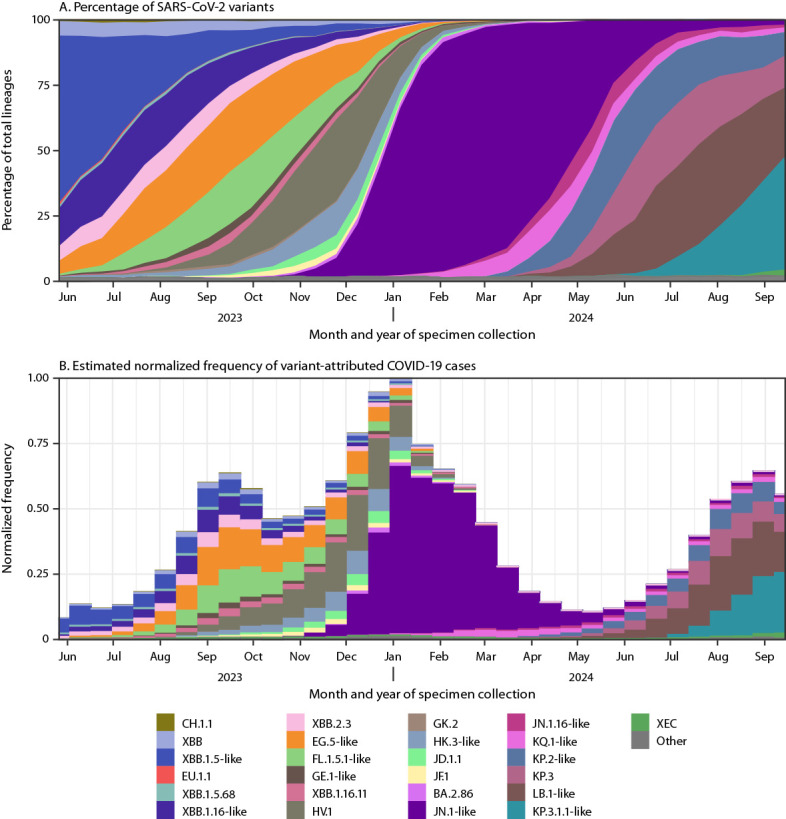
National estimates of biweekly proportions* of SARS-CoV-2 variants^†^ (A) and estimated normalized frequency of variant-attributed COVID-19 cases^§^ (B) — United States, May 14, 2023–September 14, 2024 **Abbreviations:** NREVSS = National Respiratory and Enteric Virus Surveillance System; NS3 = National SARS-CoV-2 Strain Surveillance. * Sequences are reported to CDC through NS3, contract laboratories, public health laboratories, and other U.S. institutions. Variant proportion estimation methods use a complex survey design and statistical weights to account for the probability that a specimen is sequenced. https://covid.cdc.gov/covid-data-tracker/#variant-proportions ^†^ Lineages reaching a prevalence of ≥1% with spike protein substitutions of potential therapeutic relevance and separated out on the COVID Data Tracker website (https://covid.cdc.gov/covid-data-tracker/#variant-proportions). Lineages were ordered by date of first appearance on the COVID Data Tracker. Lineages with identical spike residue 31 and receptor binding domain amino acid sequences (residues 332–527) were grouped with a representative lineage and denoted as “representative lineage-like.” “Other” represents aggregated lineages circulating at <1% prevalence nationally during all 2-week periods displayed. ^§^ Normalized frequency of COVID-19 cases attributable to variants was estimated by multiplying biweekly counts of positive test results from NREVSS with variant proportions and scaling by the maximum NREVSS case count, which occurred in the 2-week period ending January 6, 2024.

During May 14, 2023–September 14, 2024, all SARS-CoV-2 lineages circulating at ≥1% prevalence remained Omicron descendant lineages. The variant landscape during summer and fall 2023 was characterized by cocirculation of XBB descendants (Figure 1), many of which independently acquired identical substitutions in the spike protein receptor binding domain. Relative to XBB.1.5, EG.5-like lineages acquired the F456L substitution; FL.1.5.1-like lineages, HV.1 lineage, and HK.3-like lineages also contained the F456L substitution and also acquired K478R, L452R, and L455F substitutions, respectively (Table).

**TABLE:** Predominant amino acid deletions and substitutions in the receptor binding domain (residues 332–527) and residue 31 of the spike protein relative to XBB.1.5* among Omicron lineage groups with ≥5% prevalence^†^ — United States, May 14, 2023–September 14, 2024

Lineage	Spike protein amino acid substitutions																			
	31^§,¶^	332	346^§,^**	356	368	403	445**	450**	452^§,^**	455^§,^**	456^§,^**	475**	478	481	483	484**	486^§,^**	490**	493**	521
Reference sequence: XBB.1.5*	S	I	T	K	I	R	P	N	L	L	F	A	K	N	V	A	P	S	Q	P
XBB	—^††^	—	—	—	—	—	—	—	—	—	—	—	—	—	—	—	S	—	—	—
XBB.1.16-like (HF.1, XBB.1.16, XBB.1.16.1, and XBB.1.16.17)	—	—	—	—	—	—	—	—	—	—	—	—	R	—	—	—	—	—	—	—
XBB.2.3	—	—	—	—	—	—	—	—	—	—	—	—	—	—	—	—	—	—	—	S
EG.5-like (EG.5, EG.6.1, FD.1.1, FE.1.1, XBB.1.5.10, XBB.1.5.59, and XBB.1.5.72)	—	—	—	—	—	—	—	—	—	—	L	—	—	—	—	—	—	—	—	—
FL.1.5.1-like (FL.1.5.1 and XBB.1.16.6)	—	—	—	—	—	—	—	—	—	—	L	—	R	—	—	—	—	—	—	—
HV.1	—	—	—	—	—	—	—	—	R	—	L	—	—	—	—	—	—	—	—	—
HK.3-like (EG.5.1.8, GK.1.1, HK.3, JG.3, and XBB.1.5.70)	—	—	—	—	—	—	—	—	—	F	L	—	—	—	—	—	—	—	—	—
JD.1.1	—	—	—	—	—	—	—	—	—	F	L	V	—	—	—	—	—	—	—	—
JN.1-like (JN.1, JN.1.13, JN.1.32, JN.1.7, JN.1.8.1, JN.1.4.3, KV.2, and XDP)	—	V	R	T	L	K	H	D	W	S	—	—	—	K	—	K	—	F	—	—
JN.1.16-like (JN.1.11.1, JN.1.16, KW.1.1, KP.1.2, and XDV.1)	—	V	R	T	L	K	H	D	W	S	L	—	—	K	—	K	—	F	—	—
KQ.1-like (JN.1.13.1, JN.1.18, and KQ.1)	—	V	—	T	L	K	H	D	W	S	—	—	—	K	—	K	—	F	—	—
KP.2-like (JN.1.16.1, KP.1.1, KP.2, KS.1, KP.4.1, and LF.3.1)	—	V	—	T	L	K	H	D	W	S	L	—	—	K	—	K	—	F	—	—
KP.3 (XEC^§§^)	—	V	R	T	L	K	H	D	W	S	L	—	—	K	—	K	—	F	E	—
LB.1-like (KP.1.1.3, KP.2.3, LB.1, and LP.1)	Δ	V	—	T	L	K	H	D	W	S	L	—	—	K	—	K	—	F	—	—
KP.3.1.1-like (MC.1)	Δ	V	R	T	L	K	H	D	W	S	L	—	—	K	—	K	—	F	E	—

**Abbreviations: **A = alanine; C = cysteine; D = aspartic acid; Δ = deletion; E = glutamic acid; F = phenylalanine; G = glycine; H = histidine; I = isoleucine; K = lysine; L = leucine; M = methionine; N = asparagine; P = proline; Q = glutamine; R = arginine; RBD = receptor binding domain; S = serine; T = threonine; V = valine; W = tryptophan; Y = tyrosine.

* The XBB.1.5 spike protein sequence was used as a reference because of its inclusion in 2023–2024 COVID-19 vaccines. Substitutions present relative to Wuhan-Hu-1 are underlined. XBB.1.5-like lineages also include FD.2, XBB.1.42.2, XBB.1.5, XBB.1.5.1, XBB.1.9.1, and XBB.1.9.2.

^†^ Lineages with identical spike residue 31 and RBD (residues 332–527) amino acid sequences were grouped with a representative lineage and denoted as “representative lineage-like.” Lineage groups with ≥5% prevalence in at least one 2-week period and substitutions present in ≥50% of sequences belonging to a lineage were included. Lineages were ordered by date of first appearance on CDC’s COVID Data Tracker.

^§^ Indicates sites of independent substitutions in at least two different evolutionary lineages.

^¶^ Residue 31 in the N-terminal domain of the spike protein is included because of multiple independent acquisition of deletions in different descendant lineages of JN.1.

** Indicates sites identified in a previous study (https://www.nature.com/articles/s41586-021-04385-3) associated with in vitro reductions in binding by monoclonal antibodies that were previously Food and Drug Administration–authorized.

^††^ Dashes indicate the same amino acid as the reference sequence.

^§§^ XEC is a recombinant of JN.1 lineages KS.1.1 and KP.3.3 and contains the same RBD sequence as KP.3. XEC also has substitutions T22N and F59S.

None of these XBB lineage groups attained predominance (>50% prevalence), but five groups reached a prevalence of ≥10%. Prevalence of XBB.1.16-like lineages exceeded 10% by the 2-week period ending May 27, 2023, followed by EG.5-like lineages by June 24, FL.1.5.1-like lineages by August 5, HV.1 by September 30, and HK.3-like lineages by November 11 (Figure 1). The prevalence of these lineage groups peaked at 21.2% for XBB.1.16-like lineages by July 8, 2023; 26.2% for EG.5-like lineages by September 16; 20.1% for FL.1.5.1-like lineages by September 30; 12.5% for HK.3-like lineages and 31.1% for HV.1 lineage by November 25. A relative increase in COVID-19 cases occurred in late summer and early fall 2023 as these lineages cocirculated (Figure 1).

### JN.1 Predominance

**FIGURE 2:**
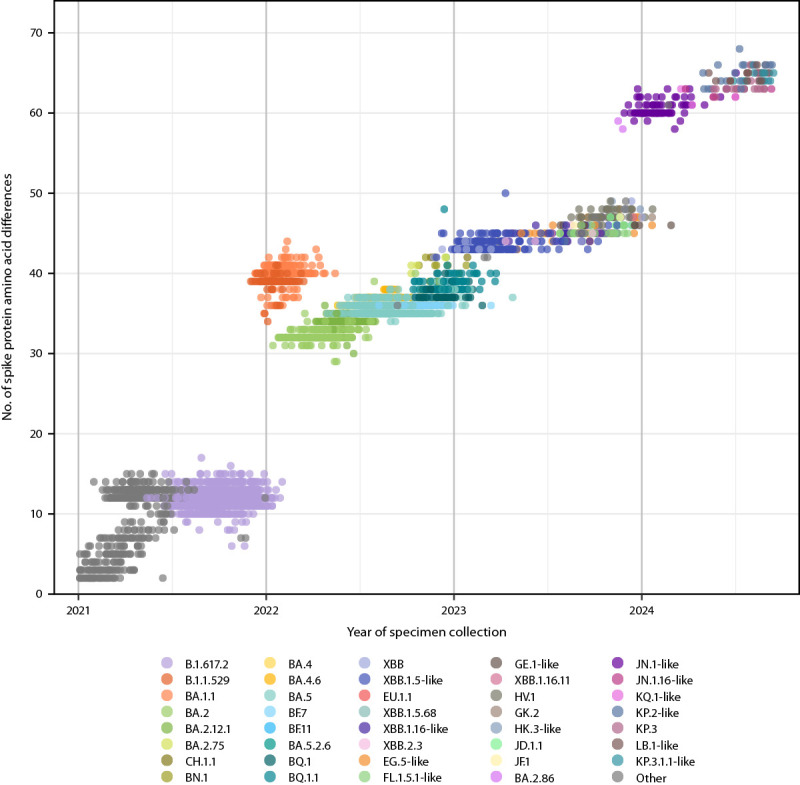
Subsampled* SARS-CoV-2 sequences,^†^ by lineage group,^§^ date of specimen collection, and number of spike protein amino acid differences (including substitutions, insertions, and deletions) relative to Wuhan-Hu-1 reference — United States, January 1, 2021–September 14, 2024 * Sequences were subsampled (5,000) for analysis from an initial dataset of 1 million sequences spanning January 1, 2021–September 14, 2024. Each year within the study period was proportionately represented, and subsampling accounted for geographic representation by ensuring that sequences from each state were included. ^†^ Sequences are reported to CDC through the National SARS-CoV-2 Strain Surveillance program, contract laboratories, public health laboratories, and other U.S. institutions. ^§^ Lineages reaching a prevalence of ≥1% with spike protein substitutions of potential therapeutic relevance and separated out on the COVID Data Tracker website (https://covid.cdc.gov/covid-data-tracker/#variant-proportions). Lineages were ordered by date of first appearance on the COVID Data Tracker. Lineages with identical spike residue 31 and receptor binding domain amino acid sequences (residues 332–527) were grouped with a representative lineage and denoted as “representative lineage-like.” “Other” represents aggregated lineages circulating at <1% prevalence nationally during all 2-week periods displayed.

BA.2.86 was reported in the United States in August 2023 through CDC’s national genomic surveillance program and other complementary surveillance systems.^§§§§^ BA.2.86 is a highly divergent descendant of BA.2 with >30 spike protein amino acid differences when compared with XBB.1.5, which is similar to the genetic distance from Delta (B.1.617.2) to Omicron BA.1.1 (Figure 2). BA.2.86 prevalence has remained <3% prevalence; JN.1, a descendant of BA.2.86 containing the L455S substitution, increased rapidly during late 2023 and was predominant nationally during January 6–April 27, 2024 (Figure 1). Increases in prevalence were similar across HHS regions, although predominance was attained slightly earlier on December 23, 2023, in Region 2 (New Jersey, New York, Puerto Rico, and the U.S. Virgin Islands). National predominance of JN.1 coincided with the highest frequency of positive SARS-CoV-2 test results reported to NREVSS during May 2023–September 2024, although other lineages were predominant during the first half of the winter surge (Figure 1). The rate of increase in prevalence of JN.1was similar to that of XBB.1.5 after reaching 1% prevalence, but was slower compared with Omicron BA.1.1 and BA.5 (Supplementary Figure 3, https://stacks.cdc.gov/view/cdc/165774).

### JN.1 Descendants

In spring and summer 2024, multiple descendants of JN.1 acquired spike protein substitutions convergently and began increasing in prevalence (Figure 1). Relative to JN.1, JN.1.16-like lineages contain the F456L substitution, KQ.1-like lineages contain R346T, KP.2-like lineages contain both substitutions, and KP.3 contains F456L and Q493E (Table). LB.1-like lineages and KP.3.1.1 acquired a deletion outside the spike receptor binding domain at residue 31 and increased in prevalence during May–September 2024 (Figure 1). KQ.1-like and KP.2-like lineages each reached >10% prevalence by April 13, 2024, KP.3 and LB.1-like lineages by May 25, and KP.3.1.1 by July 20. The prevalence of XEC, a recombinant of JN.1 lineages KS.1.1 and KP.3.3 (Table), increased from 0.4% on August 17 to 2.3% on September 14. Normalized frequencies of positive SARS-CoV-2 test results increased during late summer 2024 as these descendants of JN.1 circulated (Figure 1).

## Discussion

During May 2023–September 2024, SARS-CoV-2 lineages reported by CDC’s genomic surveillance program primarily comprised descendants of XBB and JN.1. Parallels in the evolutionary trajectories of these two lineages and their descendants were observed. The detections of XBB and BA.2.86 in fall 2022 and late summer 2023 (*5*), respectively, represented a substantial genetic shift, but XBB and BA.2.86 had relatively limited spread. These lineages subsequently acquired key spike substitutions (S486P in XBB leading to XBB.1.5 and similar lineages and L455S in BA.2.86 leading to JN.1) (*6*); by January of the following years, XBB.1.5 and JN.1 became predominant nationwide until late spring. The shift to JN.1 was followed by increased COVID-19 activity in summer 2024 and cocirculation of descendant lineages with identical substitutions, including the S31 deletion, R346T, and F456L (*7*,*8*), similar to trends after XBB.1.5 predominance. Continued monitoring to determine whether this pattern of divergent variant emergence followed by subsequent stepwise evolutionary changes continues will be important for updating COVID-19 vaccines and anticipating surges in COVID-19 activity.

### Limitations

The findings in this report are subject to at least four limitations. First, the precision of recent SARS-CoV-2 variant proportion estimates might be low because of limited data and potential biases in specimen collection or sequencing. Second, current analyses might differ from previous analyses because of changes in data sources and methods. Third, difficulties exist in ascertaining whether shifts in predominant SARS-CoV-2 variants drive COVID-19 epidemic surges, or whether increases in infection allow new variants to emerge and become predominant. Finally, decreases in sample sizes have reduced estimate precision and frequency, underscoring the need for sustainable surveillance data sources. Sensitivity analyses indicate current estimates are qualitatively similar to previous periods with larger sample sizes (*3*). The power of this platform to detect emerging variants can be maintained with continued submission of sequences from representatively sampled specimens, including during periods of low overall COVID-19 activity.

### Implications for Public Health Practice

National genomic surveillance was essential for detecting the emergence of JN.1 and other variants in the United States and demonstrating that SARS-CoV-2 continues to undergo large genetic shifts. However, surveillance data indicate that the consequences of these changes on rates of COVID-19 hospitalization and death have been reduced (*9*), likely because of widespread immunity to SARS-CoV-2.^¶¶¶¶^ Nonetheless, careful genomic monitoring remains important, as highlighted by evidence suggesting diminished 2023–2024 COVID-19 vaccine protection against JN.1 hospitalization (*2*). Data on variant proportions were used by the Food and Drug Administration to recommend inclusion of JN.1 lineages (preferentially KP.2) in updated 2024–2025 COVID-19 vaccines and are expected to guide composition of future vaccines (*10*). Given the unpredictability of SARS-CoV-2 evolution, continued monitoring for genetic changes and the impact of those changes on COVID-19 disease severity and medical countermeasure effectiveness remains essential to maintain preparedness.

## Footnotes

* These authors contributed equally to this report.

† https://covid.cdc.gov/covid-data-tracker/#variant-proportions. Estimates from earlier weeks are available at https://data.cdc.gov/Laboratory-Surveillance/SARS-CoV-2-Variant-Proportions/jr58-6ysp.

§ World Health Organization Technical Advisory Group on COVID-19 Vaccine Composition statement (https://www.who.int/news/item/26-04-2024-statement-on-the-antigen-composition-of-covid-19-vaccines); Food and Drug Administration statement (https://www.fda.gov/vaccines-blood-biologics/updated-covid-19-vaccines-use-united-states-beginning-fall-2024).

¶ https://www.cdc.gov/coronavirus/2019-ncov/variants/cdc-role-surveillance.html; https://www.aphl.org/programs/infectious_disease/SARS-CoV-2/Pages/Sequence-Based-Surveillance-Submission.aspx

** Public sequence data repositories included the Global Initiative on Sharing All Influenza Data and National Center for Biotechnology Information GenBank databases. Sequences from public repositories are limited to those meeting baseline surveillance criteria, which ensures that they correctly record geographic, demographic, and clinical diversity. https://www.aphl.org/programs/infectious_disease/Documents/Technical%20Assistance%20for%20Categorizing%20Baseline%20Surveillance%20Update%2008112021_final.pdf

†† Quality filters included limiting sequences to human-derived sources and United States–specific sequences and excluding those with invalid state names and laboratory sources. Pango lineage definitions and methods are available online. https://github.com/cov-lineages/pango-designation; https://cov-lineages.org/

§§ Because of limited precision, variant proportions from HHS regions with fewer than 300 sequences in a 2-week period are not reported on CDC’s COVID Data Tracker. https://www.hhs.gov/about/agencies/iea/regional-offices/index.html

¶¶ For estimates on CDC’s COVID Data Tracker within the most recent 4 weeks when numbers of available sequences are still accumulating, Nowcast estimates were conducted using multinomial regression fit on the previous 21 weeks of data for any lineages with ≥0.5% prevalence. In this analysis, data were limited to sequences collected beyond the most recent 4 weeks.

*** Variant proportion estimation methods account for the complex survey design of sampling, with weights based on the weekly estimated number of infections represented by each SARS-CoV-2 sequence; weights are trimmed to the 99th percentile. Each submitting laboratory source was considered a primary sampling unit, and the state and week of sequence specimen collection were considered strata. Code and model equations for variant proportion estimation methods are available online. https://github.com/CDCgov/SARS-CoV-2_Genomic_Surveillance

††† NREVSS is a network of approximately 540 laboratories that passively report weekly aggregated numbers of tests performed and results that were positive, including for SARS-CoV-2. https://www.cdc.gov/nrevss/php/dashboard/index.html

§§§ During May 11, 2023–November 17, 2023, a combination of data sources was used to estimate survey design weights. The percentage of positive SARS-CoV-2 nucleic acid amplification test results by HHS region was obtained from NREVSS, and the number of positive specimens by HHS region was obtained from COVID-19 electronic laboratory reporting (CELR). Percentages of positive test results from NREVSS and CELR correlated well. https://doi.org/10.15585/mmwr.mm7219e2

¶¶¶ Sequences were subsampled (5,000) for analysis from an initial dataset of 1 million sequences during January 1, 2021–September 14, 2024. Each year within the study period was proportionately represented and subsampling accounted for geographic representation by ensuring that sequences from each state were included.

**** 45 C.F.R. part 46.102(l)(2), 21 C.F.R. part 56; 42 U.S.C. Sect.241(d); 5 U.S.C. Sect.552a; 44 U.S.C. Sect. 3501 et seq.

†††† SARS-CoV-2 sequences originated from the 50 U.S. states, District of Columbia, American Samoa, Guam, Northern Mariana Islands, Puerto Rico, and U.S. Virgin Islands.

§§§§ CDC uses a diverse, multicomponent surveillance approach to track the emergence of new and potentially significant SARS-CoV-2 variants across the United States and globally. These surveillance systems include 1) national SARS-CoV-2 genomic surveillance, 2) traveler-based genomic surveillance, 3) the National Wastewater Surveillance System, and 4) digital public health surveillance (e.g., global data repositories, news, and social media). https://covid.cdc.gov/covid-data-tracker/#variant-summary

¶¶¶¶ Data on COVID-19 hospitalizations, deaths, and seroprevalence, an indicator of previous SARS-CoV-2 infection or vaccination, are available on CDC’s COVID Data Tracker at https://covid.cdc.gov/covid-data-tracker/#covidnet-hospitalization-network, https://covid.cdc.gov/covid-data-tracker/#trends_weeklydeaths_select_00, and https://covid.cdc.gov/covid-data-tracker/#nationwide-blood-donor-seroprevalence-2022.
